# Sunitinib in the therapy of malignant paragangliomas: report on the efficacy in a SDHB mutation carrier and review of the literature

**DOI:** 10.1590/2359-3997000000217

**Published:** 2016-09-26

**Authors:** Letizia Canu, Silvia Pradella, Elena Rapizzi, Rossella Fucci, Andrea Valeri, Vittorio Briganti, Valentino Giachè, Gabriele Parenti, Tonino Ercolino, Massimo Mannelli

**Affiliations:** 1 Department of Experimental and Clinical Biomedical Sciences University of Florence Florence Italy Department of Experimental and Clinical Biomedical Sciences, University of Florence, Florence, Italy; 2 Department of Diagnostic Radiology 2 Azienda Ospedaliera-Universitaria Careggi Florence Italy Department of Diagnostic Radiology 2, Azienda Ospedaliera-Universitaria Careggi, Florence, Italy; 3 Azienda Ospedaliera-Universitaria Careggi Florence Italy General and Surgical Unit, Azienda Ospedaliera-Universitaria Careggi, Florence, Italy; 4 Azienda Ospedaliera-Universitaria Careggi Florence Italy Division of Nuclear Medicine, Azienda Ospedaliera-Universitaria Careggi, Florence, Italy; 5 Azienda Ospedaliera-Universitaria Careggi Florence Italy Endocrinology Unit, Azienda Ospedaliera-Universitaria Careggi, Florence, Italy

## Abstract

Metastatic pheochromocytomas (PHEOs) and paragangliomas (sPGLs) are rare neural crest-derived tumors with a poor prognosis. About 50% of them are due to germ-line mutations of the SDHB gene. At present, there is no cure for these tumors. Their therapy is palliative and represented by different options among which antiangiogenic drugs, like sunitinib, have been hypothesized to be effective especially in malignant SDHB mutated tumors. We report the effects of sunitinib therapy in a SDHB mutation carrier affected by a malignant sPGL. During 101 weeks of therapy at different doses, sunitinib was able to cause a partial response and then a stable disease for a total of 78 weeks. This favorable response is the longest, out of the 35 so far reported in the literature, registered in a patient treated exclusively with sunitinib but, similarly to the other responses, the effect was limited in time. From our analysis of the scanty data present in the literature, the effect of sunitinib does not seem to be different among wild-type patients and those carrying a cluster 1 germ-line mutation. Sunitinib seems able to slow the disease progression in some patients with malignant PHEO/PGL and therefore may represent a therapeutic option, although randomized controlled studies are needed to assess its efficacy definitively in the treatment of these aggressive tumors.

## INTRODUCTION

Pheochromocytomas (PHEOs) and paragangliomas (PGLs) are neural crest-derived tumors ( 1 ). They are benign in about 90% of cases. Malignancy is diagnosed in the presence of metastases in organs devoid of chromaffin tissue such as bones, lymph nodes, liver and lungs.

Malignancy rate mostly depends on genetic background; about 50% of malignant PHEO/PGL are due to a germ-line mutation in the *SDHB* gene ( 2 - 3 ).

Patients with benign PHEOs/sPGLs are cured by the surgical removal of the tumor while the treatment of malignant PHEOs/PGLs is palliative and aimed at prolonging patient survival and/or improving patient’s quality of life ( 4 ). In the presence of a metastatic PHEO/PGL, 5 year survival is about 50% ( 5 - 7 ).

Treatment of patients with malignant PHEOs/sPGLs stems on several options: surgery, when feasible, is generally performed on the primary tumor and is mostly aimed at limiting the effects of high levels of catecholamines on target organs and, if radionuclide therapy is programmed, at enhancing I^131^-metaiodobenzylguanidine (MIBG) uptake by the remaining metastatic lesions ( 8 ). Radionuclide therapy using somatostatin analogs tracers has seldom proven to be effective ( 9 ). Chemotherapy has been employed mostly in progressive disease, with partial success, combining different drugs such as cyclophosphamide, vincristine and dacarbazine (CVD) ( 10 ) or with the alkylating drug temozolomide causing stable disease in up to 50% of the cases ( 11 ).

More recently other compounds have been proposed. Among these, drugs such as sunitinib have been hypothesized to be effective in *SDHB* mutated PGL in view of their genetic profile, characterized by an activation of the angiogenic pathway ( 12 , 13 ).

In this paper, we report on the effect obtained by sunitinib, administered as monotherapy, in a *SDHB* mutation carrier affected by a metastatic PGL and review the literature reporting the response to sunitinib in similar cases.

## CASE REPORT

A 35 year old Caucasian male with a metastatic abdominal paraganglioma was referred to our Unit in September 2013. The patient, presenting a congenital right kidney hypoplasia, had already undergone surgery twice: at the age of 10 years, when a PGL localized near the left kidney was surgically removed, and at the age of 31 when he was operated for a local recurrence.

In 2012 he started presenting symptoms of catecholamine excess like hypertensive crises, palpitations and headache. At that time, urinary normetanephrine (NMNu) was reported to be elevated. A ^18^FDG-PET showed persistent disease at the primary site and uptake in the left ischium. A I^123^MIBG scintigraphy resulted positive only at the bone level. A ^111^-In-Pentetrotide scan (Octreoscan) showed a low density of somatostatin receptors.

In September 2013, at admission, the laboratory tests showed a very high level of NMNu (8927 mcg/24h). A CT scan showed a new large abdominal recurrence 46x49x59 mm in size, located in the left lumbar-aortic region, other smaller abdominal peritoneal lesions (maximum diameter 20 mm) as well as several liver metastases.

A ^99^Tc-diphosphonate bone scintigraphy resulted negative.

After written informed consent the patient underwent genetic testing, including all the major susceptibility genes. A heterozygous G>A transversion variant at position +1 of intron 4 was found in the *SDHB* gene.

Despite doxazosine therapy at the dose of 2 mg/day, the patient blood pressure resulted 140/105 mmHg. Therefore, doxazosin dosage was progressively increased until normotension was obtained.

In October 2013, compression of the left ureter by the abdominal mass caused hydronephrosis and a sharp increase in serum creatinine (2.40 mg/dL). The obstruction was resolved by a pigtail insertion.

**Figure 1:**
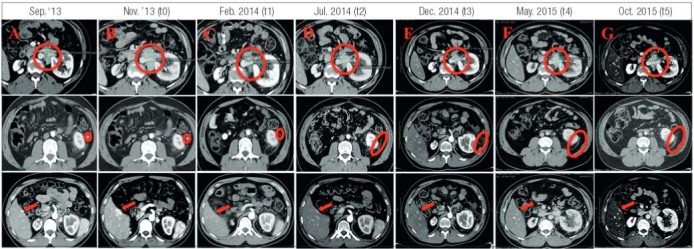
CT scan before sunitinib therapy: Sept. ’13 (A) and Nov.’13 (B), and during the follow up: t1 (C), t2 (D), t3 (E), t4 (F) and t5 (G). In the first line the main abdominal lesion, in the second line another abdominal lesion and in the third line liver metastases.

**Figure 2:**
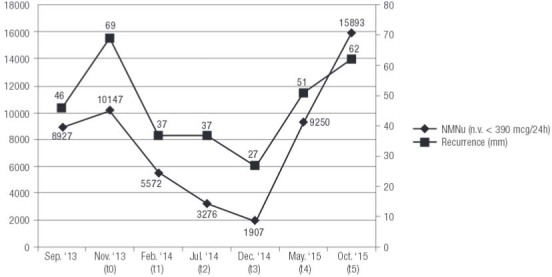
Trend of urinary normetanephrine (NMNu) combined with the size of tumor recurrence (mm).

After two months (November 2013, t0), without any anticancer therapy, a CT scan showed a significant increase in size of the main lesion (69x56x77 mm) ( Figure 1B ). NMNu resulted further increased (10147 mcg/24h) ( Figure 2 ). In view of a disease progression (PD), the patient started Sunitinib therapy.

Disease progression was evaluated during the follow up by RECIST criteria (version 1.1) ( 14 ). The main abdominal recurrence was considered the target lesion.

**Figure 3:**
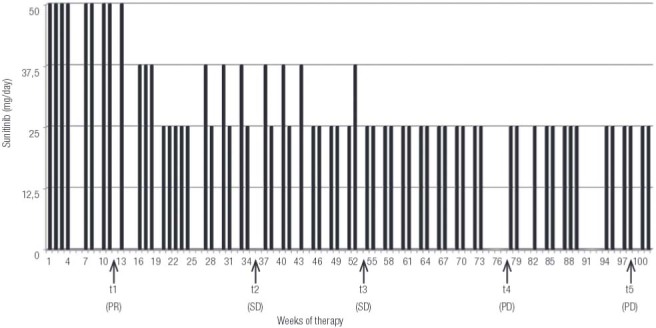
Therapy schedule from November 2013 (t0) to November 2015 (t5).

Sunitinib doses and time schedules are reported in Figure 3 . Both time schedules and doses were in time adjusted to the maximal time length and drug doses accepted by the patient, depending on the drug induced side effects ( Table 1 ).

**Table 1:** Side effects evaluated by the Common Toxicity Criteria Manual version 2.0. Grade 0 no adverse event or within normal limits; grade 1 mild adverse event; grade 2 moderate adverse event; grade 3: severe and undesirable adverse event; grade 4 life-threatening or disabling adverse event; grade 5 death related to adverse event

Side effects	Drugs	Grades of adverse events
Fatigue	-	3
Stomach pain with nausea and vomiting	Ranitidine and ondansetron	3
Hypothyroidism	Levothyroxine	1
Hypertriglyceridemia	ω 3 and fenofibrate	2
Hypertension	Doxazosin, calcium antagonist	2
Sore mouth	Mouthwash with aloe or baking soda	3

**Figure 4:**
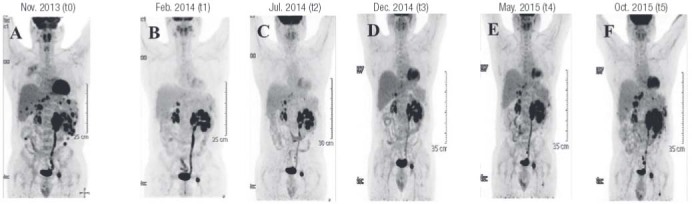
18FDG-PET before (A) and during (B-F) sunitinib therapy.

In February 2014 (t1), at first follow up, a partial response (PR) as documented by a reduction in size of the main abdominal lesion (37x35x36 mm) and the other abdominal and liver metastases ( Figure 1C ), as well as a significant decrease in NMNu levels (5572 mcg/24h) were found ( Figure 2 ). The ^18^FDG-PET scan showed a reduction in the uptake of the liver metastases and in the number and uptake of the abdominal lesions. The uptake in the main abdominal lesion didn’t change significantly ( Figure 4B ).

In July 2014 (t2), after additional 5 months of therapy, the disease was found stable at CT (main abdominal lesion: 37x38x36 mm) (SD) ( Figure 1D ) and ^18^FDG-PET ( Figure 4C ) while NMNu levels continued to decline (3276 mcg/24h) ( Figure 2 ).

At the third follow up in December 2014 (t3), the main abdominal lesion was further decreased in size (27x34x34 mm) while the other abdominal lesions and the liver metastases resulted unchanged ( Figure 1E ).

The ^18^FDG-PET scan showed a lower uptake of liver metastases and no change in the bone lesion ( Figure 4D ). The levels of NMNu were 1907 mcg/24h ( Figure 2 ). In May 2015 (t4), at the fourth follow up, the patient presented PD on CT ( Figure 1F ) and ^18^FDG-PET ( Figure 4E ). The main abdominal lesion had increased in size (51x38x60 mm) and two new peritoneal lesions appeared while the liver metastases resulted unchanged. According to these findings, NMNu was found increased to 9250 mcg/24h ( Figure 2 ).

In October 2015 (t5), after almost two years of therapy with Sunitinib, we observed a further increase in the size (62x50x75 mm) of the main abdominal lesion and in the number of liver metastases ( Figure 1G ) lately confirmed by ^18^FDG-PET ( Figure 4F ).

To date the patient has completed 101 weeks of therapy and has PD, according to the RECIST criteria 1.1. He’s continuing Sunitinib therapy at low doses (25 mg/day 2 weeks on, 1 week off) with limited side effects. Radiosurgery on the primary tumor and the liver and bone metastatic lesions is ongoing.

## DISCUSSION

In this paper we report the long lasting effect of sunitinib in a patient affected by a metastatic PGL. The surgical option on the primary lesion was discarded in view of the congenital right renal hypoplasia and the close adhesion of the tumor mass to the left kidney and ureter causing a high risk of postsurgical chronic renal failure. Radionuclide therapy with radiolabeled MIBG ( 15 ) or somatostatin analogs ( 16 ) was impeded by the insufficient uptakes of both the compounds. Therefore, we decided to start medical therapy using sunitinib.

Sunitinib is an oral tyrosine kinase inhibitor that targets the signaling pathways of VEGF receptors 1 and 2, PDGF-β receptor, and other tyrosine kinases (c-KIT, FLT3, and RET) ( 17 ).

The therapy schedule recommended in the treatment of advanced renal cell carcinoma or gastrointestinal stromal tumors is 50 mg/day 4 weeks on and 2 weeks off ( 21 ), but it is generally accepted to adapt it to patient’s tolerability. Therefore, in time, we reduced the doses and changed the drug schedule according to the side effects, mainly gastric pain and sore mouth, that the patient complained of. Thus, during the treatment, the patient’s performance status was maintained grade 2 according to ECOG performance status criteria ( 18 ).

After an initial PD in the absence of therapy, Sunitinib caused a PR, as evaluated by RECIST criteria, lasting 3 months followed by a period of SD lasting 10 months.

In the following 12 months, we observed a slight progressive increase in the sizes of the primary lesion that nevertheless were still lower than those measured at the start of sunitinib administration (69 mm *vs* 62 mm). As a whole, sunitinib slowed the disease progression in the last two years, allowing the patient to have a fairly good quality of life. It is also possible that the slight progression observed in the last 12 months might depend on the reduction in the drug doses, decided to limit its side effects.

To our knowledge, at present, only other 35 patients affected by a malignant PHEO/sPGL and treated with sunitinib have been reported in the literature ( 12 - 13 , 18 - 28 ). Their characteristics, as well as those of our patient are reported in Table 2 .

**Table 2:** Summary of the literature review.

Author	Age at the time of diagnosis	Tumor	Genetic analysis	Surgery before sunitinib	Treatment	Wk of therapy	Outcome
Park KS and cols., 2009	M (17 yr)	PHEO	NA	Yes	37,5 mg/day for 7 weeks and 25 mg/day for 4 weeks	11	PR* after 7 weeks (according to ^18^FDG uptake) followed by SD* after 11 weeks
Jimenez C and cols., 2009	F (32 yr)	PHEO (10.5 cm)	VHL	Yes	50 mg/day 4 weeks on, 2 weeks off	36	PR*
Joshua AM and cols., 2009	M (55 yr)	Abdominal PGL (14.4 cm)	SDHB	No (after six cycles)	50/mg day 4 weeks on, 2 weeks off (before surgery); 37.5 mg/ day 4 weeks on, 2 weeks off (after surgery)	48	PR after 36 weeks followed by PD after 48 weeks (+ surgery)
	M (28 yr)	Abdominal PGL (7 cm)	SDHB	Yes	50/mg day 4 weeks on, 2 weeks off**	40	PR
	F (41 yr)	PHEO (15 cm)	Negative for SDHB, SDHD, RET and VHL	Yes	50 mg/day 4 weeks on, 2 weeks off**	40	PR*
Hahn NM and cols., 2009	F (33 yr)	Abdominal PGL (17 cm)	SDHB	Yes	50 mg/day 4 weeks on, 2 weeks off; 50 mg/day 2 weeks on, 1 week off	16	PD*
Cirillo F, 2010	M (37 yr)	Abdominal PGL (17x14x9 cm)	NA	Yes	50 mg/day 4 weeks on, 2 weeks off; 25 mg/day 4 weeks on, 2 weeks off; 25 mg/day 2 weeks on, 1 week off	24	SD* after 15 weeks followed by PD* after 24 weeks (+ octreotide LAR)
Zukauskaite R and cols., 2011	M (31 yr)	PGL thoracic-lumbar region (10x15 cm)	No somatic mutations	Yes	50 mg/day 4 weeks on, 2 weeks off	24	SD* after 12 weeks followed by PD* after 24 weeks
	F (54 yr)	PHEO	Sporadic	Yes	50 mg/day 4 weeks on, 2 weeks off reduced up to 12.5 mg/day	68	SD* after 40 weeks followed by PD* after 68 weeks
Ayala-Ramirez M and cols., 2012 F (8); M (9)	(33 yr)	PHEO	VHL	No	50 mg/day 4 weeks on, 2 weeks off or 37.5 mg/day continously or 37.5 mg/day 3 weeks on, 1 week off	24	SD
	(60 yr)	PHEO	Sporadic	No	50 mg/day 4 weeks on, 2 weeks off or 37.5 mg/day continously or 37.5 mg/day 3 weeks on, 1 week off	44	PR
	(55 yr)	PGL	SDHB	No	50 mg/day 4 weeks on, 2 weeks off or 37.5 mg/day continously or 37.5 mg/day 3 weeks on, 1 week off	108	SD
	(20 yr)	PGL	SDHB	No	50 mg/day 4 weeks on, 2 weeks off or 37.5 mg/day continously or 37.5 mg/day 3 weeks on, 1 week off	NA	SD
	(62 yr)	PHEO	Sporadic	No	50 mg/day 4 weeks on, 2 weeks off or 37.5 mg/day continously or 37.5 mg/day 3 weeks on, 1 week off	1.6	PD
	(14 yr)	PHEO	Sporadic	No	50 mg/day 4 weeks on, 2 weeks off or 37.5 mg/day continously or 37.5 mg/day 3 weeks on, 1 week off	13	PD
	(47 yr)	PHEO	Sporadic	No	50 mg/day 4 weeks on, 2 weeks off or 37.5 mg/day continously or 37.5 mg/day 3 weeks on, 1 week off	16	PD
	(40 yr)	PHEO	Sporadic	No	50 mg/day 4 weeks on, 2 weeks off or 37.5 mg/day continously or 37.5 mg/day 3 weeks on, 1 week off	4	PD
	(57 yr)	PGL	SDHB	No	50 mg/day 4 weeks on, 2 weeks off or 37.5 mg/day continously or 37.5 mg/day 3 weeks on, 1 week off	NA	NA (sunitinib was stopped due to toxicity)
	(60 yr)	PGL	SDHB	No	50 mg/day 4 weeks on, 2 weeks off or 37.5 mg/day continously or 37.5 mg/day 3 weeks on, 1 week off	NA	NA (sunitinib was stopped due to toxicity)
	(69 yr)	PHEO	Sporadic	No	50 mg/day 4 weeks on, 2 weeks off or 37.5 mg/day continously or 37.5 mg/day 3 weeks on, 1 week off	NA	NA (sunitinib was stopped due to toxicity)
	(27 yr)	PHEO	SDHB	No	50 mg/day 4 weeks on, 2 weeks off or 37.5 mg/day continously or 37.5 mg/day 3 weeks on, 1 week off	NA	SD
	(56 yr)	PHEO	Sporadic	No	50 mg/day 4 weeks on, 2 weeks off or 37.5 mg/day continously or 37.5 mg/day 3 weeks on, 1 week off	48	PR
	(45 yr)	PGL	SDHB	No	50 mg/day 4 weeks on, 2 weeks off or 37.5 mg/day continously or 37.5 mg/day 3 weeks on, 1 week off	18	PR
	(40 yr)	PGL	SDHB	No	50 mg/day 4 weeks on, 2 weeks off or 37.5 mg/day continously or 37.5 mg/day 3 weeks on, 1 week off	32	SD
	(43 yr)	PHEO	SDHB	No	50 mg/day 4 weeks on, 2 weeks off or 37.5 mg/day continously or 37.5 mg/day 3 weeks on, 1 week off	16.4	PD
	(63 yr)	PHEO	Sporadic	No	50 mg/day 4 weeks on, 2 weeks off or 37.5 mg/day continously or 37.5 mg/day 3 weeks on, 1 week off	8.4	PD
Nemoto K and cols., 2012	F (41 yr)	PHEO (10 cm)	NA	Yes	50 mg/day 4 weeks on, 2 weeks off; 25 mg/day 2 weeks on, 2 weeks off	26	PR
Sun FK and cols., 2012	M (32 yr)	PHEO (18 cm)	Negative for SDHB, SDHD, RET and VHL	Yes	50 mg/day 4 weeks on, 2 weeks off; 37.5 mg/day 4 weeks on, 2 weeks off	22	Enlargement in the necrosis area of tumor with SD*
	M (51 yr)	PHEO (12.9 cm)	Negative for SDHB, SDHD, RET and VHL	Yes	50 mg/day 4 weeks on, 2 weeks off	28	Necrosis of the lesions at the CT scan (PR*)
	F (49 yr)	PHEO (5 cm)	Negative for SDHB, SDHD, RET and VHL	Yes	50 mg/day 4 weeks on, 2 weeks off	30	PR*
Prochilo T and cols., 2012	F (35 yr)	Abdominal PGL	SDHB	Yes	50 mg/day 4 weeks on, 2 weeks off; 37.5 mg daily 2 weeks on, 2 weeks off; 25 mg daily 2 weeks on, 1 week off	More than 36	PR* after 12 weeks followed by SD* after 36 weeks and finally PD* (evaluated by ^18^FDG-PET)
Hata J and cols., 2014	M (23 yr)	PHEO (8.7 cm)	NA	Yes	50 mg/day 4 weeks on, 2 weeks off	20	SD (the authors not reported how many weeks after) followed by PD* after 20 weeks
	M (60 yr)	PHEO (7.2 cm)	NA	Yes	50 mg/day 4 weeks on, 2 weeks off**	16	SD* (the authors not reported how many weeks after) followed by PD* after 16 weeks
Lebowitz-Amit R and cols., 2014	M (51 yr)	Abdominal PGL (6.9x5.9 x 7.1 cm)	Negative for SDHB, SDHC, SDHD, TMEM127 and NF1	Yes	50 mg/day; 37.5 mg/day; 25/37.5 mg/day alternating	24	SD*
Bourcier ME and cols., 2013	F (70 yr)	Abdominal PGL	NA	Yes	50 mg/day 4 weeks on, 2 weeks off	12	CR
Our case	M (35yr)	Abdominal PGL	SDHB	yes	25 mg/day 2 weeks on, 1 week off for the of time	101	PR after 12 weeks followed by SD after 32 weeks up to 54 weeks and PD after 78 weeks

F: female; M: male; NA: not available; PGL: paraganglioma; PHEO: pheochromocytoma; * data deducted by the case description and not by RECIST evaluation; ** deducted data. Weeks are always reported from the beginning of sunitinib therapy.

In this series 20 patients were males and 16 females, aging from 14 to 70 yrs.

Genetic analysis was performed in 30 out of 36 patients. 15 patients resulted wild-type, 2 Von Hippel Lindau ( *VHL* ) mutation carriers and 13 *SDHB* mutation carriers. Therefore, a *SDHB* germ-line mutation was found in 43.3% (13/30) of genotyped patients, in agreement with the high frequency of malignancy reported in *SDHB* mutation carriers ( 29 ).

Surgery on the primary tumor had been carried out in 50% of patients (18/36). Sunitinib was administered as first non surgical therapy in 33% (12/36).

The length of sunitinib therapy has been reported in 31/36 patients and ranges from 16 to 108 weeks.

The outcome of sunitinib therapy has been reported according to different criteria: in 19 patients the outcome has been calculated by RECIST criteria while in 14 patients the outcome has been reported by unspecified criteria. In 3 patients the outcome was not reported as the treatment was interrupted shortly after the start of therapy because of drug toxicity.

As a whole, 7 patients experienced PD, while sunitinib resulted effective in 72.2% of patients (26/36), providing a complete response (CR) in 1 patient, a PR in 13 patients and a SD in 12 patients.

CR was observed after 12 weeks. In all patients (13/13) with PR the length of therapy is reported and the drug effect was maintained after a period ranging from 11 to 101 weeks. In this group, PD latterly ensued in 3 patients, after 48, more than 36 and 78 weeks from the beginning of therapy.

Among the 12 patients found with SD the length of therapy was reported in ten of them, ranging from 16 to 108 weeks and 5 were reported to undergo PD at different times after the beginning of therapy (from 16 to 68 weeks).

It has been hypothesized that the genetic background might affect the effects of antiangiogenic therapy, resulting more effective in PHEO/PGL belonging to cluster 1 ( 30 ).

When analyzing this series reported in the literature, among the 30 genotyped patients, the outcome was reported in 14 wild type (wt) patients and in 13 mutation carriers (11 SDHB and 2 VHL). PR or SD was reported in 9 wt and 11 mutated patients, respectively. Therefore, at least from this limited series, the different genetic background does not seem to affect sunitinib efficacy.

In summary, from the scant data of the literature, sunitinib seems able, in some patients, to slow the progression of the disease and its efficacy does not seem to depend on tumor genetic background. Nevertheless, its real efficacy will be stated only after the results of proper controlled studies. At present, only two such studies are ongoing in patients with malignant PHEOs/sPGLs: the FIRSTMAPPP study (First International Randomized Study in Malignant Progressive Pheochromocytoma and Paraganglioma) and the SNIPP study (Study of Sunitinib in Patients with Recurrent Paraganglioma/Pheochromocytoma). Both are Phase II studies.

FIRSTMAPPP is a multicenter and randomized study (sunitinib 37.5 mg/day *versus* placebo) while SNIPP is a non randomized study (sunitinib 50 mg/day 4 weeks on, 2 weeks off).

In conclusion, we describe the case of a patient with malignant PHEO treated only with sunitinib for a very long period (101 weeks). The drug was able to induce a PR after 12 weeks and to maintain its effects (SD) for an additional 66 weeks. In spite of a slow disease progression, at present the patient still presents a good quality of life. Because of PD, the patient has started treatment with Temozolomide.

The results we observed in our patient are similar to those reported in other patients similarly affected by malignant PHEO/PGL. As a whole, sunitinib seems to offer a therapeutic option in some of these patients, although its effect seems limited in time.
